# EEG Cortical Connectivity Analysis of Working Memory Reveals Topological Reorganization in Theta and Alpha Bands

**DOI:** 10.3389/fnhum.2017.00237

**Published:** 2017-05-12

**Authors:** Zhongxiang Dai, Joshua de Souza, Julian Lim, Paul M. Ho, Yu Chen, Junhua Li, Nitish Thakor, Anastasios Bezerianos, Yu Sun

**Affiliations:** ^1^Centre for Life Science, Singapore Institute for Neurotechnology, National University of SingaporeSingapore, Singapore; ^2^Neuroscience and Behavioral Disorders Program, Centre of Cognitive Neuroscience, Duke-NUS Graduate Medical SchoolSingapore, Singapore; ^3^Computational Intelligence Lab, School of Computer Engineering, Nanyang Technological UniversitySingapore, Singapore

**Keywords:** cortical functional connectivity, EEG, eLORETA, graph theory, *n*-back, working memory

## Abstract

Numerous studies have revealed various working memory (WM)-related brain activities that originate from various cortical regions and oscillate at different frequencies. However, multi-frequency band analysis of the brain network in WM in the cortical space remains largely unexplored. In this study, we employed a graph theoretical framework to characterize the topological properties of the brain functional network in the theta and alpha frequency bands during WM tasks. Twenty-eight subjects performed visual *n*-back tasks at two difficulty levels, i.e., 0-back (control task) and 2-back (WM task). After preprocessing, Electroencephalogram (EEG) signals were projected into the source space and 80 cortical brain regions were selected for further analysis. Subsequently, the theta- and alpha-band networks were constructed by calculating the Pearson correlation coefficients between the power series (obtained by concatenating the power values of all epochs in each session) of all pairs of brain regions. Graph theoretical approaches were then employed to estimate the topological properties of the brain networks at different WM tasks. We found higher functional integration in the theta band and lower functional segregation in the alpha band in the WM task compared with the control task. Moreover, compared to the 0-back task, altered regional centrality was revealed in the 2-back task in various brain regions that mainly resided in the frontal, temporal and occipital lobes, with distinct presentations in the theta and alpha bands. In addition, significant negative correlations were found between the reaction time with the average path length of the theta-band network and the local clustering of the alpha-band network, which demonstrates the potential for using the brain network metrics as biomarkers for predicting the task performance during WM tasks.

## Introduction

Working memory (WM) is a type of memory system that enables temporary storage and processing of information (Baddeley, 2003, 2012; Postle, 2006). It allows for a limited amount of information to be held and manipulated in the mental workspace. WM is a critical module as it provides the basis for higher-level cognitive functions, and plays an essential role in many daily activities such as problem solving, decision-making, and the acquisition of new skills (Gathercole and Baddeley, 1993; Pickering, 2006; Logie, 2014). For example, as revealed by the Ease of Language Understanding (ELU) model (Rönnberg et al., 2013), WM provides crucial support for language processing and speech perception.

Different WM-related tasks impose various levels of demand/load on the WM system. A major topic in the study of WM has been on how the brain characteristics are modulated by the presence of or changes in memory loads, which is induced by WM tasks with different difficulty levels (Cappell et al., 2010; Michels et al., 2012; Konstantinou and Lavie, 2013). Various electroencephalogram (EEG) studies have revealed the involvement of the theta- and alpha-band activities in WM. Specifically, the activity in the theta band, particularly in the frontal lobe (Grunwald et al., 2014; Hsieh and Ranganath, 2014; Roux and Uhlhaas, 2014), has been consistently found to be positively correlated with WM demand and to be a reliable indicator of variations in the memory load of WM tasks (Sauseng et al., 2005; Langer et al., 2013; Grunwald et al., 2014). The positive correlation has been explained by the fact that theta oscillation is responsible for coordinating and integrating different cognitive processes during the execution of WM tasks, which leads to heightened theta activity during high-demand WM tasks due to the active recruitment of cognitive resources (Sarnthein et al., 1998; Sauseng et al., 2010). On the other hand, alpha-band oscillation has been discovered to be related to the inhibition of the brain activities that are not involved in the mental task (Klimesch et al., 2007; Mazaheri and Jensen, 2010; Scheeringa et al., 2012; Uusberg et al., 2013), which has led to its negative association with the amount of mental resources employed during a cognitive task and the memory load in WM tasks (Smith et al., 1999; Gevins et al., 2012; Roux et al., 2012). These studies may be considered compatible with evidence on the two specialized storage systems for WM information (Baddeley, 2012) and indicate distinct functional roles and anatomical regions involved in the generation of theta/alpha-band oscillations (Roux and Uhlhaas, 2014).

Despite these promising findings, most of these EEG studies were conducted in sensor space, leading to possible bias in inferring the locations of the sources inside the brain that are responsible for the observed activity on the scalp (Schoffelen and Gross, 2009; Ewald et al., 2012). Although other neuroimaging techniques such as functional magnetic resonance imaging (fMRI) could significantly improve the spatial resolution, these methods are constrained by low temporal resolution and therefore unable to reveal the multi-frequency aspects of the WM-related characteristics of the brain activity. In order to inspect the activity of the brain with both high temporal and spatial resolutions, several recent studies of WM have been performed in EEG source space, in which the sources of the corresponding brain activities are localized through source localization techniques (Gevins et al., 1997; Tuladhar et al., 2007; Palva et al., 2010; Langer et al., 2013). It is noteworthy that most EEG studies of WM so far have adopted univariate approaches, such as evaluating the changes in the spectral power of different frequency bands (Sauseng et al., 2005; Scheeringa et al., 2009; Escolano et al., 2011; Gevins et al., 2012; Grunwald et al., 2014), and the event-related potentials (ERPs; Duarte et al., 2013; Missonnier et al., 2013; Dong et al., 2015; Katus et al., 2015). These studies mainly focused on the regional properties of the brain, largely neglecting the brain characteristics from a global perspective. Therefore, a comprehensive approach in the EEG source space that adequately reflects the overall organization of the brain has the potential of shedding more light on the mechanism of WM processing.

Accumulating studies have revealed that cognition emerges from the interactions among various brain regions that may be spatially separated but functionally and/or structurally linked (Rubinov and Sporns, 2010; Park and Friston, 2013). Drawing inspiration from this underlying mechanism of the brain, a computational framework examining the brain as a complex network (Bullmore and Sporns, 2009; Rubinov and Sporns, 2010) has the potential of providing more extensive understanding of WM. The human brain can be represented as a “connectome,” or a large-scale network of interconnected regions, which provides the anatomical substrate for neural communication, functional processing and information integration (Sporns, 2011). Functional connections are constructed by estimating the statistical associations between the activities of different pairs of brain regions. The brain functional connectivity network, which unfolds dynamically upon the rigid structural connectivity network across multiple scales, closely reflects the internal state of the brain activities (Park and Friston, 2013). In recent years, numerous studies on the brain have been conducted through complex network analysis methods (Sporns and Zwi, 2004; Bullmore and Sporns, 2009; Rubinov and Sporns, 2010). Using graph theoretical models, which represent the brain regions as nodes and the functional/structural connections as edges, various neurobiologically meaningful measures have proved instrumental in examining the characteristics of the cortical network (Rubinov and Sporns, 2010).

The application of graph theoretical analysis to EEG functional connectivity network has led to significant research findings (Micheloyannis et al., 2006; de Haan et al., 2009). Specifically, convergent evidence has demonstrated that human brain networks possess a special topological organization, i.e., small-world architecture, which is characterized by the combination of dense local clustering of neighboring nodes and short path lengths between distant nodes (Watts and Strogatz, 1998). This ideal combination is believed to provide an optimal brain structure that simultaneously supports locally segregated and globally integrated processing (Bassett and Bullmore, 2006, 2009; Sporns, 2011). In previous studies of brain functional connectivity network, we have applied a similar graph theoretical analytical framework in characterizing various cognitive mental states (Sun et al., 2014a, 2017) and neurological disorders (Sun et al., 2014b), which revealed that the topology of the brain network could be modulated by mental fatigue (mainly characterized by increased average path length among brain regions) as well as Alzheimer's disease (mainly manifested by reduced efficiency of local information transfer). A recent study in the EEG source space, which also utilized graph theoretical analysis, discovered training effects of WM on the functional connectivity network (Langer et al., 2013). The current study on the effects of WM load, which employs a similar analytical framework, made some methodological modifications by utilizing a more accurate source localization technique, performing the analysis in two different frequency bands and exploring more comprehensive graph theoretical metrics.

In this study, EEG data was recorded during *n*-back tasks (Krieger et al., 2005; Ravizza et al., 2005; Valera et al., 2010) at two difficulty levels [0-back (control task) vs. 2-back (WM task)] and transformed into the source space using the exact Low Resolution Electromagnetic Tomography (eLORETA; Pascual-Marqui et al., 2011). Subsequently, using a graph theoretical framework, we explored the topology of the cortical functional connectivity network in different frequency bands. Given that the theta (4–7 Hz) and alpha (8–12 Hz) bands have been consistently discovered to be highly correlated with WM demand (for review, see Klimesch, 1999), we focused on these two frequency bands in the EEG analysis. Specifically, we calculated graph measures of small-world properties (e.g., clustering coefficient, characteristic path length and small-worldness), global and local efficiencies, and nodal betweenness centrality to examine (1) the nature of the WM-dependent global topological alterations of the cortical functional network during WM tasks; (2) the brain regions whose centrality within the network varies with the presence of WM load; and (3) the associations between the network characteristics and the task performance. Based on the previous discoveries, we hypothesized that the functional network constructed in different frequency bands would demonstrate distinct WM-dependent alterations. We further conjectured that different sets of brain regions in the theta and alpha bands would exhibit WM-dependent levels of centrality within the functional connectivity network.

## Materials and methods

### Subjects

Twenty-eight students (age = 21.5 ± 1.6 years, male/female = 11/17) from National University of Singapore participated in this study. All subjects were right-handed according to the Modified Edinburg Questionnaire (Oldfield, 1971), and had normal or corrected-to-normal vision. Subjects were prescreened through a short telephone interview to ensure that they met all inclusion criteria in the present study, i.e., those subjects who admitted to chronic physical or mental illness, had been diagnosed with a sleep disorder or childhood history of ADHD, or were taking long-term medication were excluded. The study was approved by the Institutional Review Board of National University of Singapore (Reference No. 13-516). Written informed consent was obtained from each of the participant after the explanation of the experimental protocol. Participants were reimbursed S$20 for their participation.

### Experimental protocols

Upon arriving at the lab, the subjects were instructed to provide self-reports of their sleep history and use of alcohol/medication over the 48 h before the experiments. Given that sleep quality would affect various cognitive domains including WM (Lim and Dinges, 2010), participants who reported sleep durations of <6.5 h on either of the two previous nights were re-arranged or excluded from further participation. Subjects who took alcohol/medication within 6 h of entering the lab were excluded from further participation. The subjects were then prepared to undergo EEG recording before performing two cognitive tasks, i.e., WM (*n*-back) task and mental arithmetic task, each at two difficulty levels. Each participant was requested to complete 2 sessions of both tasks in a pseudorandom order. Each block was around 5 min and the length of the break between the consecutive blocks was around 30 s. The entire experiment [comprising eight blocks in total (2 tasks × 2 levels × 2 sessions)] lasted ~45 min. In the current study, only the EEG data recorded during the WM task were analyzed and reported.

**Figure 1:**
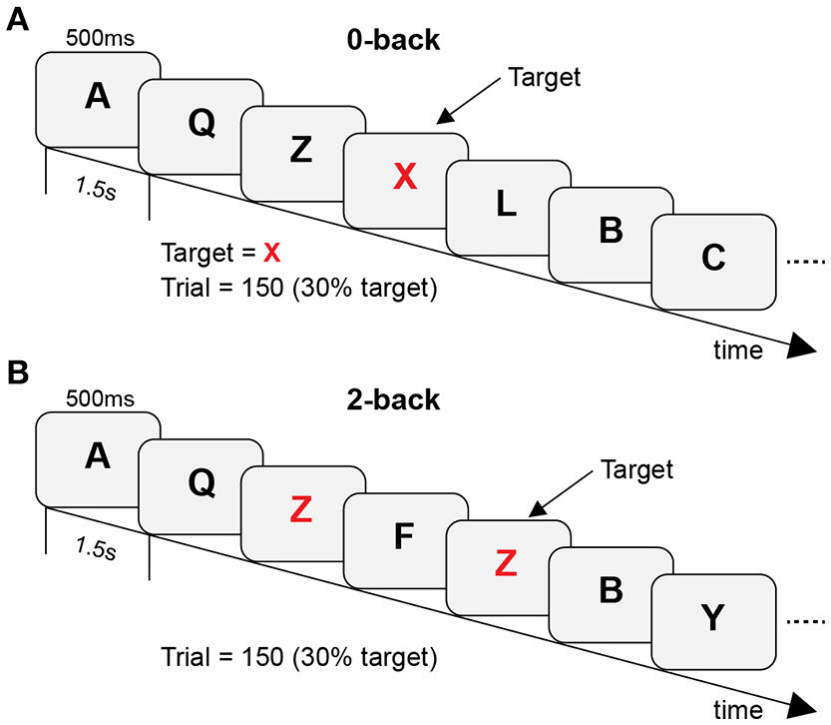
**A schematic diagram of the experimental protocol with (A)** 0-back as control tasks and **(B)** 2-back as WM tasks.

In the *n*-back task, individual uppercase letters were presented at a visual angle of ~2° × 1° in white font on a black background. Letters were presented for 500 ms with an inter-stimulus interval of 1.5 s. Stimuli were presented on a Windows computer via E-Prime 2.0 (Schneider et al., 2002). In each trial, the participants were instructed to indicate whether the current letter was a target (key-press “**P**”) or a non-target (key-press “**Q**”). Two difficulty levels of the task were created (control vs. WM tasks: 0-back vs. 2-back). In the 0-back task, the target letter was an “**X**,” and all other letters were non-targets. In the 2-back task, the targets were the letters that were identical to the letters presented two trials before it. During the experiment, behavioral data were recorded in both target and non-target conditions. Participants performed the task in blocks of 150 trials (block duration: ~5 min). Blocks were arranged in a predetermined, pseudorandom order. A schematic diagram of the experimental protocol is shown in Figure 1.

Prior to the real recordings, the participants performed one block of each of the task types to ensure that they understood the instructions. During the EEG data collection, the participants were instructed to emphasize both accuracy and speed in their performance and to avoid unnecessary head movement. The participants were scheduled individually for sessions in the afternoon (between 1 and 5 p.m.) to control for possible circadian confounds. All testing took place in the Cognitive Engineering laboratory of Singapore Institute for Neurotechnology (SINAPSE) in Singapore.

### Data acquisition

EEG data were recorded from 64-channel Ag/AgCl electrodes (including two channels from the left and right mastoids) based on the standard 10–20 system, sampled at 256 Hz with the ANT wave-guard system (ASA-Lab, ANT B.V., Netherlands), and referenced using the average reference technique. The bipolar electrooculogram (EOG) signals were recorded from the outer canthi (HEOG), and above and below (VEOG) the right eye. Electrode impedances were maintained below 10 kΩ during the entire experiment. Anti-aliasing was performed with a band-pass filter (0.5–70 Hz), and a 50 Hz notch filter was used to avoid main interferences.

### EEG data preprocessing and segmentation

**Figure 2:**
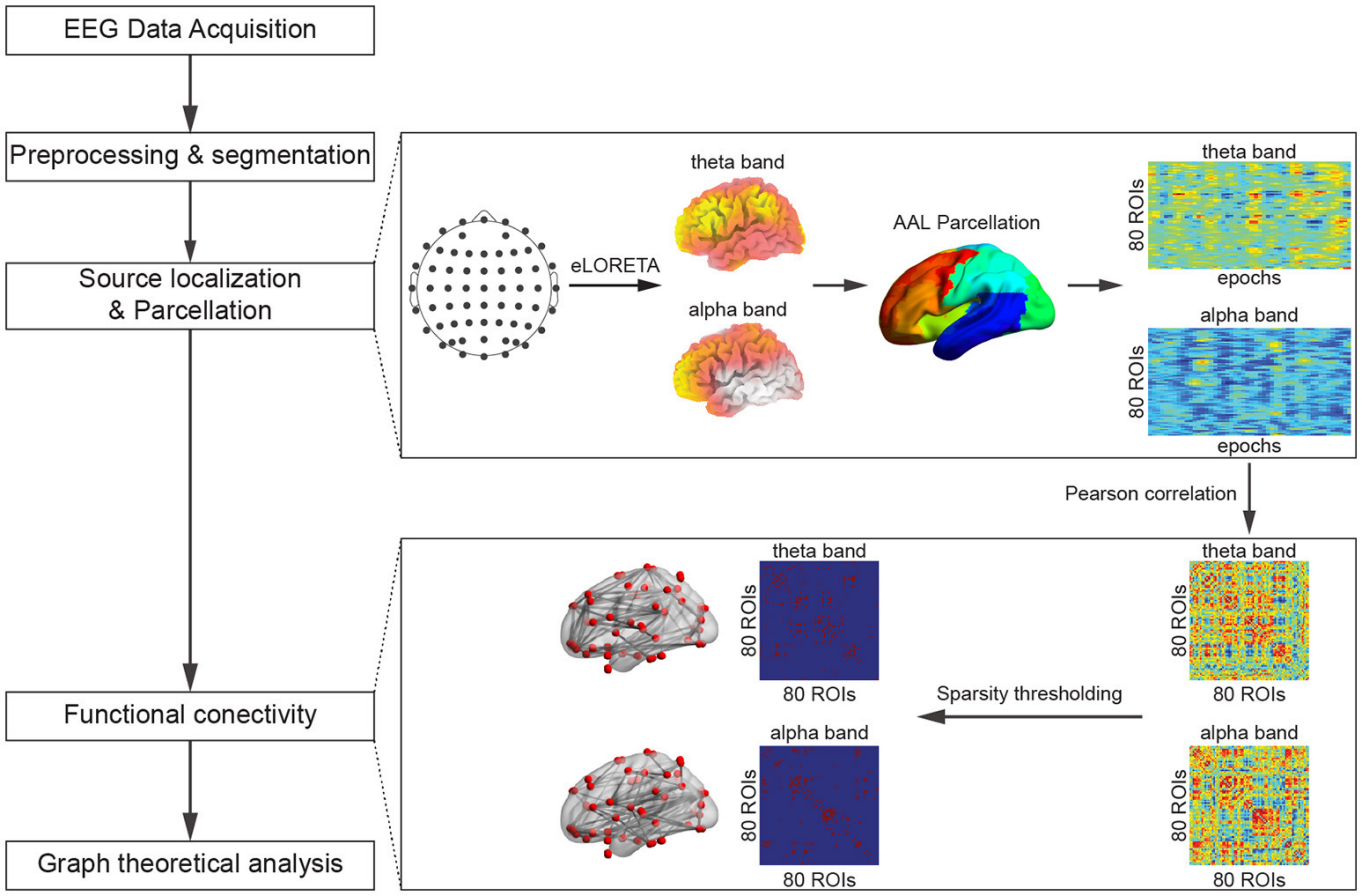
**A flowchart of the data analysis procedures**.

A flowchart that outlines the procedure of data analysis for each individual subject is presented in Figure 2.

EEG data preprocessing was performed with EEGLAB (Delorme and Makeig, 2004). The recorded EEG signals were first band-pass filtered from 1 to 40 Hz, and artifact rejection was performed through independent component analysis (ICA) in order to remove the artifacts caused by eye blinks, eye movement, muscle movement, etc. (Jung et al., 2000; James and Hesse, 2005; Mantini et al., 2008). Briefly, after ICA decomposition, the ICs were classified via the following parameters: (1) the correlation between different ICs and the horizontal as well as vertical EOG components; (2) the kurtosis k of the IC; and (3) the coefficient of determination *r*^2^ obtained by fitting the IC power spectrum with a 1/f function. An IC was classified as artifact if any of the above parameters was above a given threshold according to Mantini et al. (2009). The number of rejected ICs was different across subjects with a range of 3–8. The preprocessed EEG data were then segmented into different epochs, with the beginning of each epoch marked by the stimulus onset and the end of each epoch marked by the exact time of the subject's response in the particular trial. Therefore, the length of each EEG epoch varies across different trials and depends on the subjects' response time. Outlier detection for reaction time was performed through the boxplot, significant outlier would be removed. Here, missing responses were classified as incorrected trials and only correct target trials in each of the experimental conditions were selected for further analysis. The final number of trials were 42.30 ± 0.43 and 36.95 ± 0.87 (mean ± standard error of the mean) in the 0- and 2-back tasks respectively. No significant outlier in the number of trails was revealed using boxplot.

### Source localization

The method used here to estimate the cortical activity was similar to the procedure adopted in (Langer et al., 2013), however, we have employed a source localization technique (eLORETA) that produces more robust and accurate result (Pascual-Marqui et al., 2011; Jatoi et al., 2014) than the approach adopted in (Langer et al., 2013; sLORETA: standardized Low Resolution Electromagnetic Tomography). Specifically, for each EEG data epoch, the power spectrum of each EEG channel and the cross-coherence between all pairs of EEG channels in both frequency bands were obtained with the multi-taper approach using a Hanning window. Subsequently, eLORETA source localization was carried out using FieldTrip (Oostenveld et al., 2011) running under Matlab 2012a (Mathworks, USA). A template T1 image from the SPM8 toolbox (Litvak et al., 2011) was segmented into three tissues: scalp, brain, and skull, which were then used to create the three shell boundary element models. Electrode locations (comprising 64 channels based on the standard 10–20 system, among which the average signal of the two electrodes from the left and right mastoids were used as references to re-reference the EEG recordings) were then mapped onto the scalp using a transformation matrix obtained by FieldTrip. Tissue conductance values were kept at the default values: 0.33 S/m for the scalp and brain, and 0.008 S/m for the skull. A volume conduction model was then created based on the above criteria. Sources were restrained to the gray matter for this study, and the lead-fields were obtained using this approach. Source localization was then carried out by interpolating the sources onto the T1 image. Subsequently, the power values produced by the source localization were parcelated into 116 regions based on the Automatic Anatomical Labeling atlas (Tzourio-Mazoyer et al., 2002; by averaging the values of the voxels within each region), among which 26 cerebellar and 10 subcortical regions were excluded due to the limited capability of EEG to detect sources of electrical activity at deep locations inside the brain and in the cerebellum (Andreou et al., 2015), resulting in 80 regions of interest (ROIs) for further analysis. The names and the corresponding abbreviations of the selected cortical regions are listed in Table S1.

### Cortical functional connectivity network construction

After the source localization and parcellation, for every subject and in each frequency band, the power values derived from all epochs under the same experimental condition (0- or 2-back; one power value from each epoch) were concatenated to form a series of power values for each ROI, leading to a matrix (80 × number of correct trials). Subsequently, employing similar approaches for the estimation of cortical functional connectivity to those in Cannon et al. (2012) and Thatcher et al. (2012), the Pearson correlation coefficients between all pairs of power series were calculated, resulting in a weighted symmetric connectivity matrix (80 × 80) for every subject under each experimental condition in each frequency band.

Prior to the graph theoretical analysis, each of the obtained correlation matrices was converted into a binary matrix with a fixed sparsity value to ensure that the wiring cost of each participant were at the same level. For a given network *G* with *N* (*N* = 80) nodes, the sparsity is defined as the ratio of the number of existing edges to the maximum possible number of edges. In the current study, sparsity values in the range of 10–50% with a step of 1% were selected to maintain the network reachability and allow for prominent small-world properties in the cortical networks. All the networks with the entire sparsity range were fully connected.

### Graph theoretical analysis

In order to quantitatively investigate the topological properties of the cortical functional connectivity network in different WM tasks, we performed graph theoretical analysis on the networks using the Brain Connectivity toolbox (Rubinov and Sporns, 2010).

#### Global metrics

For a graph *G* with *N* nodes (*N* = 80 in this work), the clustering coefficient (*C*), which is a measure of the degree of local clustering of a graph, is computed as (Rubinov and Sporns, 2010):

(1) $$C=\frac{1}{N}\sum_{i\in N}\frac{2E_{i}}{\left(k_{i}\left(k_{i}-1\right)\right)},$$

where *k*_*i*_ is the number of edges directly connected with node *i*, and *E*_*i*_ is the number of triangles around node *i*. The characteristic path length, which quantifies the overall communication efficiency between any pair of nodes, is calculated as (Rubinov and Sporns, 2010):

(2) $$L=\frac{1}{N\left(N-1\right)}\sum_{i\in N}\sum_{i\neq j\in N}\min\left\{L_{ij}\right\},$$

where min {*L*_*ij*_} is the shortest path length between nodes *i* and *j*.

In order to characterize the small-world properties of the networks, the normalized clustering coefficient (γ = *C/C*_*rand*_) and normalized characteristic path length (λ = *L*/*L*_*rand*_) were calculated, in which *C*_*rand*_ and *L*_*rand*_ represent the average clustering coefficient and average characteristic path length of an ensemble of 100 surrogate random networks. Each of the random networks was generated from the original network by randomly rewiring the edges in the graph, while preserving the total number of nodes and edges, the degree distribution and the connectedness of the graph (Maslov and Sneppen, 2002). γ and λ can be unified into one metric: small-worldness (σ = γ/λ). A network is considered as small-world if it meets the criteria: γ >> 1 and λ ≈ 1 (Humphries et al., 2006).

In order to further characterize the small-world properties of the networks in terms of information flow, global efficiency (*E*_*global*_) and local efficiency (*E*_*local*_) were calculated. *E*_*global*_, which measures the overall efficiency of information exchange on the network and is inversely related to *L*, is computed as (Rubinov and Sporns, 2010):

(3) $$E_{global}=\frac{1}{N\left(N-1\right)}\sum_{i\neq j\in N}\frac{1}{\min\left\{L_{ij}\right\}},$$

*E*_*local*_, which is a measure of the efficiency of information transmission within the local clusters in a graph, is calculated as (Rubinov and Sporns, 2010):

(4) $$E_{local}=\frac{1}{N}\sum_{i\in N}E_{global}\left(G_{i}\right),$$

where *E*_*global*_(*G*_*i*_) is the global efficiency of *G*_*i*_, the subgraph consisting of the neighbors of node *i*.

#### Nodal metrics

In addition to evaluating the global metrics of the cortical network, we also performed nodal analysis and assessed the importance of different ROIs within the brain network by evaluating the betweenness centrality (*b*_*i*_) of each node in the network, which is defined as the fraction of all shortest paths in the network that pass through node *i* (Freeman, 1979).

### Statistical analysis

#### Statistical analysis of the integrated global and nodal metrics

In order to avoid the bias introduced by the selection of network sparsity, we integrated the global and nodal network measures over the entire sparsity range (Achard and Bullmore, 2007). Mathematically, the integrated metrics correspond to the areas under the respective metric curves. Subsequently, two-tailed paired *t*-tests were performed on all subjects in order to identify the metrics showing statistically significant differences between the 0- and 2-back tasks. The threshold for statistical significance was selected as 0.05 (*p* = 0.05) for the global metrics and 0.01 (*p* = 0.01) for the nodal metric. To address the problem of multiple comparison in the nodal analysis, false discovery rate (FDR) correction with a threshold of *q* = 0.05 was performed on the integrated betweenness centrality values.

#### Correlation between the behavioral metrics and integrated graph measures

In order to investigate the association of the task performance with the characteristics of the functional connectivity network and assess the capability of the network metrics for predicting the individual performance of the WM tasks, we computed the Pearson correlation coefficients between the integrated graph theoretical metrics (both global and nodal metrics) and the behavioral statistics (both the reaction time and accuracy) across all subjects. Only those network metrics that showed statistically significant group difference were investigated in the correlation analysis.

## Results

### Behavioral results

As a manipulation check, we compared the reaction times and hit rates between the two WM tasks (Table 1). A clear effect of WM was revealed in both the reaction time and accuracy. Compared with the control (0-back) task, the reaction time was significantly increased for both (target and non-target) conditions (*p* < 0.01) in the WM (2-back) task, together with a significantly reduced accuracy (*p* < 0.01).

**Table 1:** **Behavioral results of the 0-back and 2-back tasks**.

**Task level**	**Reaction time (ms)**		**Accuracy (%)**	
	**Target**	**Non-target**	**Target**	**Non-target**
0-back^a^	414.4 ± 45.6	390.4 ± 54.3	94.0 ± 5.1	98.7 ± 1.7
2-back	578.5 ± 131.0	547.7 ± 138.0	82.1 ± 10.2	95.1 ± 1.9
Statistical test^b^	*t*_(27)_ = −8.40 (*p* = 5.22e^−9^)	*t*_(27)_ = −7.44 (*p* = 5.33e^−8^)	*t*_(27)_ = 6.52 (*p* = 5.39e^−7^)	*t*_(27)_ = 8.76 (*p* = 2.26e^−9^)

a *Values are expressed as mean ± SD*.

b *The p-values were obtained using paired t-tests*.

### Global network characteristics

**Figure 3:**
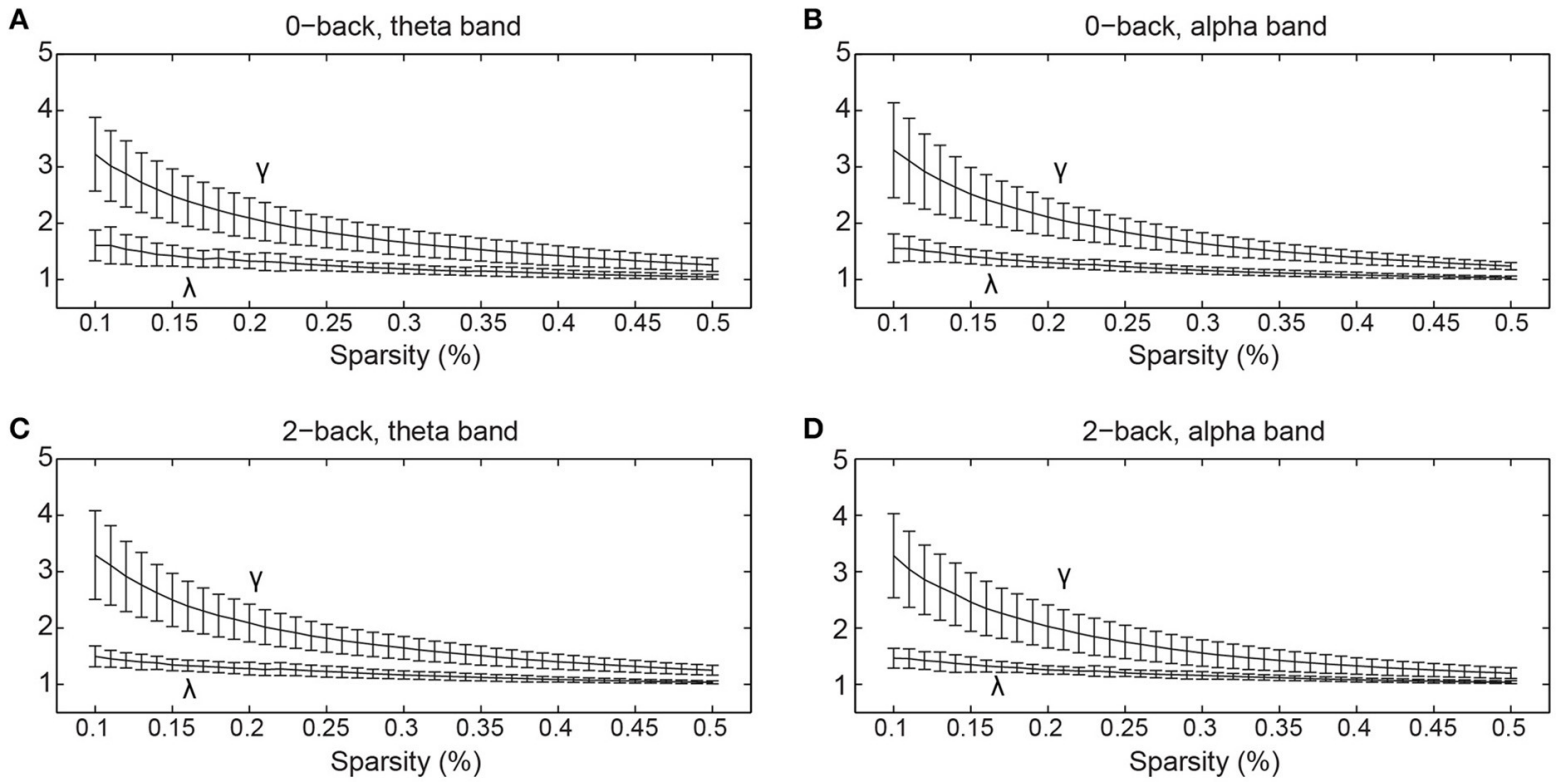
**The normalized clustering coefficient (γ) and normalized characteristic path length (λ) of the cortical functional connectivity networks over different sparsity values (mean ± standard deviation) in (A)** 0-back task, theta band, **(B)** 0-back task, alpha band, **(C)** 2-back task, theta band, and **(D)** 2-back task, alpha band.

**Figure 4:**
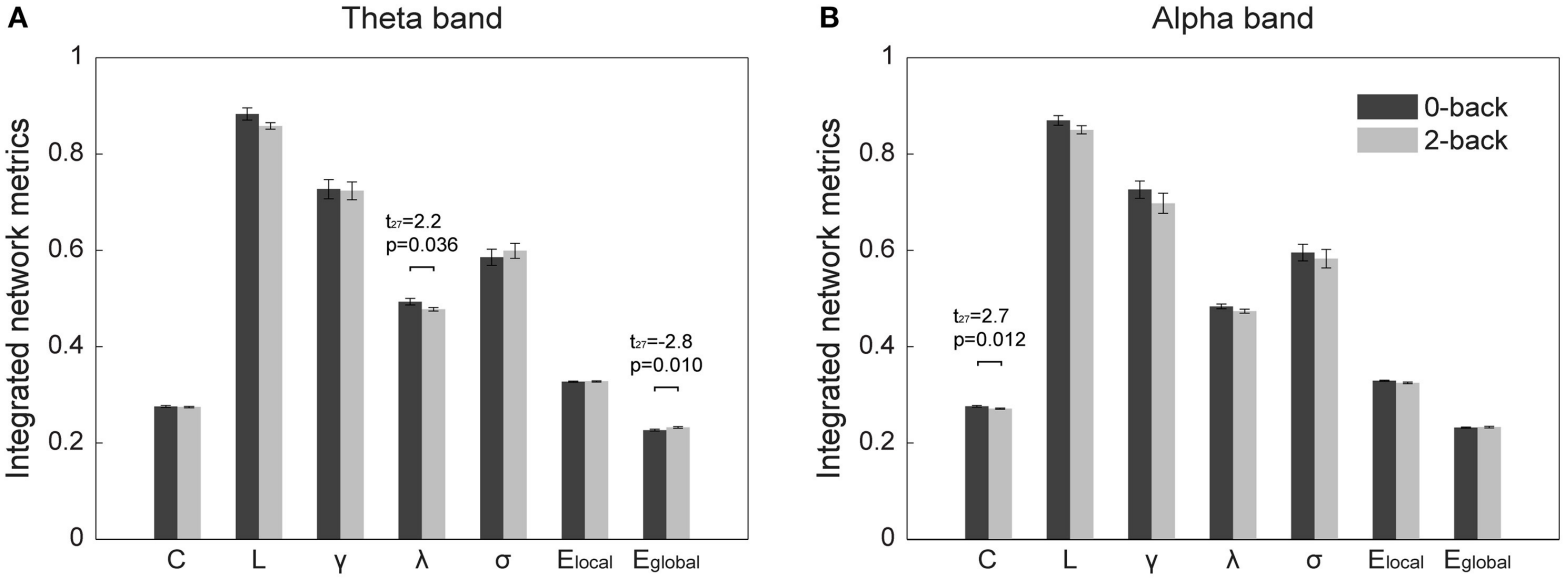
**Integrated global metrics (corresponding to the area under the curves of each metric over the entire sparsity range) of the cortical functional connectivity networks in the 0-back and 2-back tasks in (A)** theta band, **(B)** alpha band. The metrics are (from left to right) clustering coefficient (*C*), characteristic path length (*L*), normalized clustering coefficient (γ), normalized characteristic path length (λ), small-worldness (σ), local efficiency (*E*_*local*_), and global efficiency (*E*_*global*_). The bars represent mean ± standard error. The *t*- and *p*-values of the corresponding metrics showing statistically significant differences (*p* < 0.05) between the 0-back and 2-back tasks are presented.

The normalized clustering coefficient and normalized characteristic path length of the networks are shown in Figure 3. We found that the criteria for small-worldness was satisfied (γ >> 1 and λ ≈ 1) in both experimental conditions and both frequency bands. Quantitative statistical analyses revealed significant topological alterations (*p* < 0.05) in the global network metrics between the two WM tasks in both frequency bands. Figure 4 shows the integrated global metrics of the functional connectivity networks in both the theta and alpha bands. As shown in Figure 4A, in the theta-band network, compared with the 0-back task, the integrated normalized characteristic path length decreased significantly (*p* = 0.036), while the integrated global efficiency exhibited a statistically significant increase (*p* = 0.010). In the alpha-band network, as shown in Figure 4B, the clustering coefficient decreased significantly (*p* = 0.012) in the 2-back task.

### Regional network characteristics

**Figure 5:**
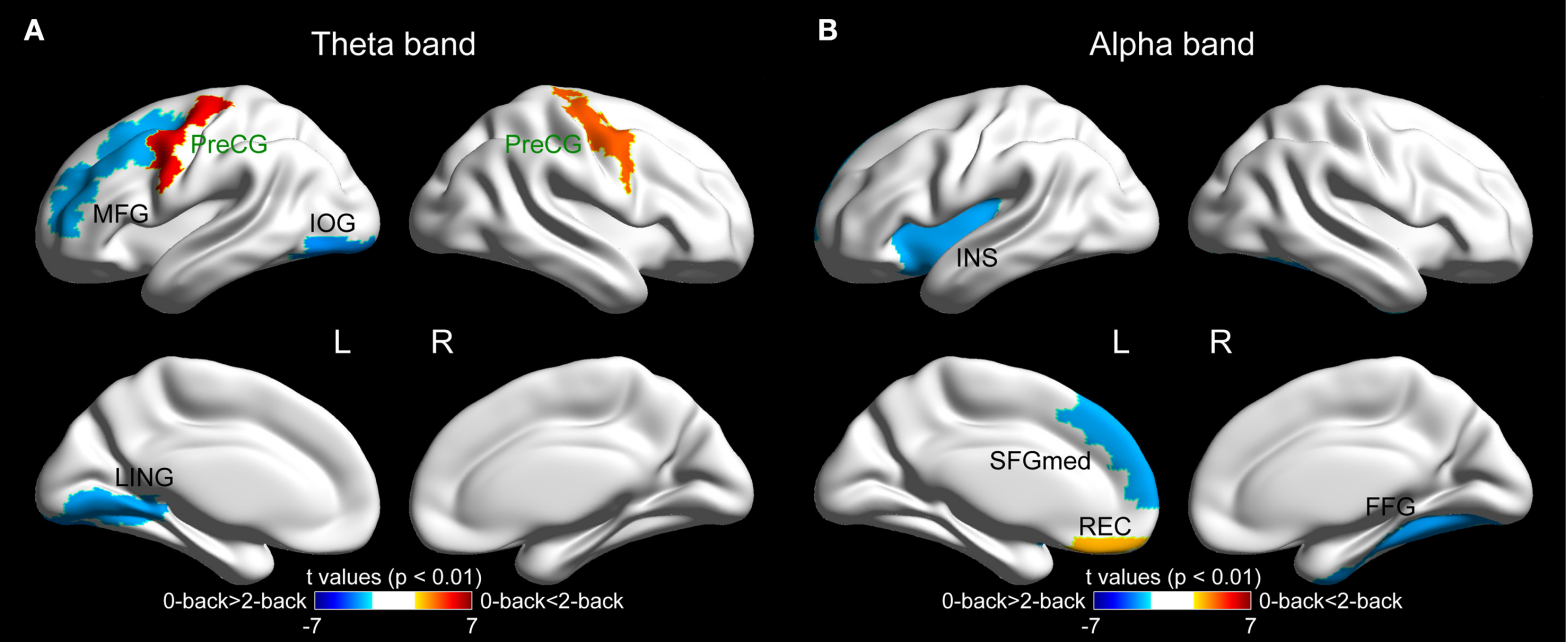
**The surface spatial distribution of the cortical regions showing significantly different betweenness centrality values between the 0-back and 2-back tasks (*p* < 0.01) in (A)** theta band and **(B)** alpha band. The color bar represents the *t*-values of the corresponding regions obtained from paired *t*-test between the two tasks. The names of the regions that survived the FDR correction are highlighted in green. The brain regions were overlaid on inflated surface maps with the BrainNet Viewer toolbox (Xia et al., 2013). For the abbreviation of the cortical regions, see Table S1. L = left, R = right.

The brain regions that showed significantly different betweenness centrality in the two experimental conditions (*p* < 0.01) are shown in Figure 5. Specifically, in the theta band, significant inter-task differences were observed in five brain regions. Three of those regions [the left middle frontal gyrus (MFG.L), *p* = 0.008, the left inferior occipital gyrus (IOG.L), *p* = 0.003 and the left lingual gyrus (LING.L), *p* = 0.006] exhibited reduced betweenness centrality values in the 2-back task, while significantly increased betweenness centrality values were revealed in the bilateral precentral gyrus (PreCG.L, *p* < 0.0001^*^, ^*^indicates the regions that survived the FDR threshold at *q* < 0.05, and PreCG.R, *p* = 0.0004^*^). In the alpha band, four brain regions showed statistically significant differences between the two tasks, among which three of them [the left insula (INS.L), *p* = 0.007, the left superior frontal gyrus, medial part, (SFGmed.L), *p* = 0.007, and the right fusiform gyrus (FFG.R), *p* = 0.005] showed reduced betweenness centrality values in the WM task, while the betweenness centrality of the left gyrus rectus (REC.L, *p* = 0.009) was increased due to the WM demand.

### Correlation between the behavioral metrics and network measures

**Figure 6:**
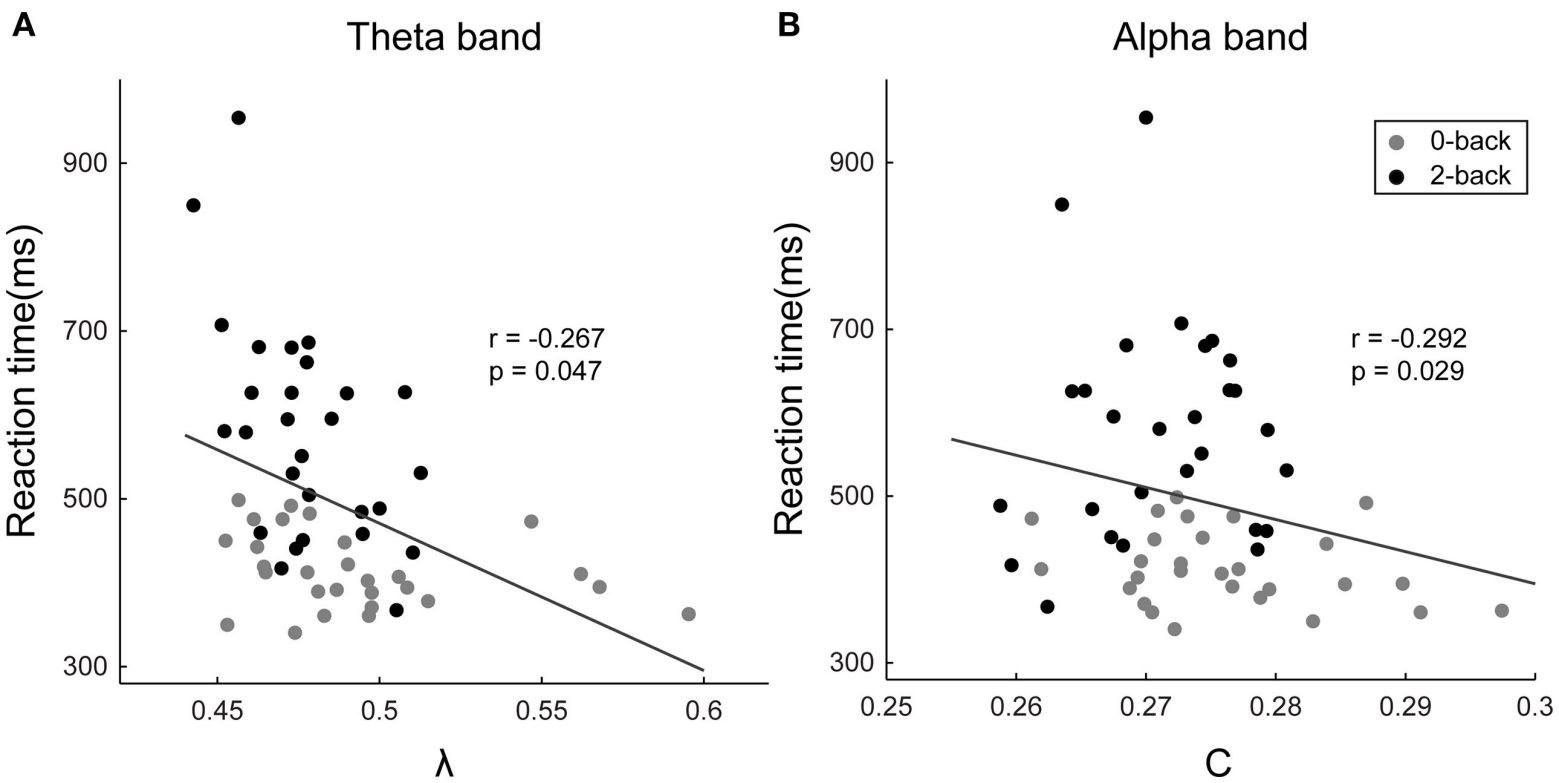
**Scatter plots showing the correlations between the subjects' reaction time and (a)** the normalized characteristic path length of the theta-band network and **(B)** the clustering coefficient of the alpha-band network. The horizontal axis represents the values of the respective graph metrics, and the vertical axis stands for the reaction time. The *r*- and *p*-values of the corresponding correlations are displayed in the figures.

The normalized characteristic path length of the theta-band network (*r* = −0.267, *p* = 0.047) and the clustering coefficient of the alpha-band network (*r* = −0.292, *p* = 0.029) were revealed to possess significant negative correlations with the reaction time (Figure 6). Moreover, a significant positive correlation was found between the betweenness centrality of the PreCG.L in the theta-band network and the reaction time (*r* = 0.340, *p* = 0.010). No statistically significant correlation was discovered between the graph metrics and the response accuracy.

## Discussion

Using a graph theoretical approach, we investigated the global and regional alterations of the cortical functional connectivity network in the theta and alpha bands in a WM task compared with a control task. We discovered that in the WM (2-back) task, the theta-band functional connectivity network became more globally integrated, leading to a more efficient overall organization; the local segregation of the alpha-band network was degraded, resulting in a less optimal network architecture. Additionally, distinct sets of brain regions in the two frequency bands exhibited WM-dependent centrality within the functional network, and regions showing both positive and negative associations with the memory demand were revealed in each frequency band. Moreover, we found significant correlations between the reaction time and the network characteristics in both frequency bands, which demonstrates the potential of the respective network characteristics for predicting the performance in the WM tasks.

### Variations in the global topology of the cortical functional connectivity network

Various studies have discovered that the brain functional network possesses small-world architecture (for reviews, see Bassett and Bullmore, 2006, 2009; Sporns, 2011), which features the combination of strong global integration and high local clustering (Watts and Strogatz, 1998). In the current study, in accordance with previous findings, the small-world topology was revealed in both task conditions (the 0- and 2-back tasks) and both frequency bands (the theta and alpha bands), which suggests that regardless of the presence of WM requirement and the frequency of oscillation, the functional connectivity network is optimally organized for efficient exchange of information. However, despite the common small-world structure, distinct alterations in the global network topology resulting from the presence of WM load were found in the theta and alpha bands.

In the theta band, the global efficiency of the cortical functional connectivity network increased significantly in the 2-back task compared with the 0-back task, while the normalized characteristic path length exhibited a significant decrease. These findings indicate enhanced functional integration of the brain network and strengthened overall interaction among different brain regions, which result in an improvement in the overall efficiency of information transfer on the brain functional network and greatly facilitate the execution of the WM task. It has been reported that the theta synchronization among different brain regions contributes substantially to the co-activation of various brain structures in WM task execution and that the theta oscillation might play an integrative role in the overall organization of the brain activity, making it actively involved in WM tasks during which various cognitive resources are recruited and coordinated (Sarnthein et al., 1998; Sauseng et al., 2010). Therefore, the observed improvement in the functional integration of the theta-band network, together with previous findings that demonstrated increased theta activity in high-load WM tasks (Gevins et al., 1997; Jensen and Tesche, 2002; Sauseng et al., 2005; Langer et al., 2013; Grunwald et al., 2014), might suggest that when a WM task (as opposed to a control task in which no WM is required) is performed, the activity of the theta-band network is enhanced toward more efficient and economical propagation of information on the network. The strengthened theta oscillation and more globally efficient network structure in the theta band might be attributed to the need for enhanced attention to facilitate the sustained maintenance of memory representations, in order to cope with the memory-demanding WM task (Gevins et al., 1997; Hsieh and Ranganath, 2014; Roux and Uhlhaas, 2014).

In the alpha-band cortical functional connectivity network, the clustering coefficient decreased significantly in the 2-back task. This observation demonstrates a decline in the functional segregation and a reduction in the local density of connections in the WM network. Many studies in recent years have led to the hypothesis that alpha oscillations are associated with the suppression of spurious brain activities and the inhibition of brain regions that are irrelevant to the mental task (Klimesch et al., 2007; Mazaheri and Jensen, 2010; Scheeringa et al., 2012; Uusberg et al., 2013), which suggests an inverse correlation between the amplitude of the alpha activity and the amount of cortical resources employed to perform the cognitive task. Specifically, previous WM studies have consistently revealed weakened alpha activity in WM tasks with increased memory demand (Cohen et al., 1997; Gevins et al., 1997, 1998, 2012; Smith et al., 1999; Roux et al., 2012; León-Domínguez et al., 2015). Therefore, taking into account the observed decline in local clustering, we speculate that this inverse relationship between the alpha activity and WM load might be associated with weakened local functional clustering of functionally related brain regions in the alpha-band network during WM tasks.

### Alterations in the importance of individual brain regions in the cortical functional connectivity network

In contrast to previous studies investigating the alterations in the activations of different brain regions in WM tasks, in the current study, we assessed the variations of the importance of individual brain regions within the cortical functional connectivity network using a graph theoretical measure: betweenness centrality.

In the theta-band functional connectivity network, three brain regions in the frontal lobe were discovered to possess WM-related betweenness centrality (the PreCG.L, PreCG.R, and MFG.L), and the activations of all three regions have previously been revealed to be associated with the load of WM tasks (Carlson et al., 1998). Of note, two out of the three regions (the PreCG.L and PreCG.R, both of which survived the FDR correction for multiple comparisons) exhibited increased importance within the brain network in the WM task. The observation, together with previous findings demonstrating heightened theta activity in the frontal regions with increased WM load (Gevins et al., 1997, 1998; Jensen and Tesche, 2002; Sauseng et al., 2005; Langer et al., 2013; Grunwald et al., 2014), might further corroborate the notion that the theta oscillations in the frontal regions become more critical for the execution of tasks requiring WM (in contrast to those tasks with the absence of WM) or with heavier WM load. Moreover, reduced centrality in the 2-back WM task was observed in two brain regions that reside in the occipital lobe (the IOG.L and LING.L). The visual cortex has been discovered to be transiently involved in the execution of WM tasks yet independent of the memory load (Cohen et al., 1997). Meanwhile, in the presence of WM load, more cognitive resources are recruited to cope with the memory-demanding WM task, and the theta oscillation has been suggested to be responsible for integrating different cognitive processes in WM (Sauseng et al., 2010). Therefore, in the theta-band network, due to the increased employment of various cognitive resources and maintained involvement of the visual cortex in WM tasks (compared with the control task), the relative participation of the visual cortex in the processing of the functional connectivity network is expected to decline, which is supported by the observation of decreased betweenness centrality of the two occipital brain regions in the current study.

In the alpha-band network, three anterior regions showed WM-dependent betweenness centrality; among these regions, two of them (the INS.L and SFGmed.L) exhibited reduced importance within the network in the WM task, while the other brain region (the REC.L) was found to be positively correlated with WM. Additionally, one posterior brain region (the FFG.R) was revealed to be negatively associated with WM. In line with our observations, the functional coupling among the anterior brain regions in the upper alpha band has been discovered to decrease with WM demand (Sauseng et al., 2005). Therefore, we speculate that the memory demand in WM tasks (compared with the control task) leads to decoupled alpha oscillations among the anterior brain regions, thus reducing the centrality of these regions within the alpha-band functional connectivity network. One of the anterior regions displaying reduced betweenness centrality in the 2-back task (the SFGmed.L) has previously been discovered to be more activated at rest than during WM tasks (Mazoyer et al., 2001). The SFGmed.L region has been consistently discovered to be contained in the default mode network (DMN; Mason et al., 2007; Uddin et al., 2009), whose activity is enhanced during the resting condition and lessened during the performance of cognitive tasks (Raichle et al., 2001; Greicius et al., 2003). Therefore, we conjecture that as a result of the presence of WM load in the 2-back task, the weakened activity of the DMN leads to the reduction in the involvement of the DMN brain regions in the alpha-band functional connectivity network and results in the observed decline in the betweenness centrality of the SFGmed.L.

### Correlations between the behavioral metrics and network measures

The performance of WM tasks, which can be quantified by behavioral measures such as reaction time, has been repeatedly found to be correlated with the functional connectivity in both theta and alpha bands (Palva et al., 2010; Roux et al., 2012; Langer et al., 2013). The normalized characteristic path length in the theta-band functional connectivity network has been found to be negatively correlated with the reaction time. Since the normalized characteristic path length is inversely associated with the global integration, this finding further corroborates the integrative role taken by the theta oscillation in coordinating different cognitive resources and the improvement in the global integration of the theta-band brain network in high-demand WM tasks (Sarnthein et al., 1998; Sauseng et al., 2010). Moreover, the clustering coefficient of the alpha-band network was revealed to possess significant negative correlation with the reaction time, which provides additional evidence in support of the inhibition of the activity of irrelevant brain regions related to alpha oscillations and the reduced functional segregation of the alpha-band network as a result of the presence of (compared with WM-free tasks) or increase in WM load (Klimesch et al., 2007; Mazaheri and Jensen, 2010; Scheeringa et al., 2012; Uusberg et al., 2013). These findings demonstrate the intimate associations between the network characteristics in different frequency bands and the task performance, and thus reveal the potential of the respective network metrics for predicting the performance of the subjects in WM tasks.

### Methodological considerations

In the present study, a standard T1 image was used in the source localization process for all subjects. We have attempted to minimize the effects of the estimation error through choosing relative large cortical parcellations and investigating the group-averaged data. However, the source localization can be improved if subject-specific anatomy were used. Therefore, future studies employing the brain anatomy of each individual subject, which could be obtained through high-resolution fMRI images, to improve the accuracy of the source localization are encouraged. Furthermore, since the current study is exploratory in nature, an uncorrected *p*-value of 0.01 was used as the threshold for establishing the significance and interpreting the results of the regional analysis. However, due to the large number of comparisons performed in the nodal analysis, the possibility that some of the nodal results may have occurred by chance cannot be ruled out. Therefore, some caution is needed when interpreting the results of the nodal analysis. In the current study, we focused on the interpretation of the general patterns of the findings; we also provided the detailed statistical results and highlighted those regions that survived the correction for multiple comparisons for readers' interpretation. Third, although EEG has several advantages in revealing neural network dynamics and the precise coordination of oscillations at different frequencies as performed in the current work, it is limited both in its spatial resolution and in its capacity to detect sources of electrical activity at deep locations and in the cerebellum (Andreou et al., 2015). Therefore, brain networks were constructed with parcellation covered only cortical areas in the current work. Future studies with cautious application of source localization methods with concurrent EEG/fMRI recordings are anticipated. Lastly, the final number of trails for functional connectivity estimation in the current study was different between control and WM tasks. Further studies with more trails and multiple load levels were needed to confirm our observations and investigate the characteristics of cortical network reorganization under various levels of WM load.

## Conclusion

In this study, we constructed cortical functional connectivity networks in the EEG source space and adopted a graph theoretical framework to analyze the topological variations of the brain network during a WM task compared with a control task. We revealed that, in the WM task (compared with the control task), the theta-band functional integration was improved, whereas the alpha-band functional segregation was reduced; the network centrality of different brain regions in the frontal, temporal and occipital regions were altered in both frequency bands; the reaction time of the subjects was negatively correlated with the average path length in the theta band and positively correlated with the degree of local clustering in the alpha band. Our findings might shed further light on the frequency band-dependent alterations in the topology of the functional brain network in WM tasks and promote our understanding of the underlying mechanism of the effects in WM.

## Author contributions

ZD, JLim, YS conceived and initiated the analysis study in this paper. PH conducted the experiment and collected the data. ZD, JD, YC, JLi, and YS analyzed the data. ZD and YS interpreted the results and drafted the manuscript. All authors reviewed the manuscript and approved the final version for the publication.

### Conflict of interest statement

The authors declare that the research was conducted in the absence of any commercial or financial relationships that could be construed as a potential conflict of interest.
