# Supplementary material for: EEG Cortical Connectivity Analysis of Working Memory Reveals Topological Reorganization in Theta and Alpha Bands
Source: Front Hum Neurosci. 2017 May 12;11:237. doi: 10.3389/fnhum.2017.00237 (PMC5427143; doi:10.3389/fnhum.2017.00237)
Supplement: Supplementary file 1 [file Table1.DOCX]

Table S1. The names and corresponding abbreviations of the regions of interest ROIs

| **Region names** | **Abbr.** | **Classes** | **Region names** | **Abbr.** | **Classes** |
| --- | --- | --- | --- | --- | --- |
| amygdala | AMYG | Paralimbic | Orbitofrontal cortex (superior) | ORBsup | Paralimbic |
| angular gyrus | ANG | Association | Orbitofrontal cortex (inferior) | ORBinf | Paralimbic |
| anterior cingulate gyrus | ACG | Paralimbic | Orbitofrontal cortex (medial) | ORBmed | Paralimbic |
| calcarine fissure | CAL | Primary | Orbitofrontal cortex (middle) | ORBmid | Paralimbic |
| cuneus | CUN | Association | paracentral lobule | PCL | Association |
| fusiform gyrus | FFG | Association | parahippocampal gyrus | PHG | Paralimbic |
| gyrus rectus | REC | Paralimbic | postcentral gyrus | PoCG | Primary |
| heschl gyrus | HES | Primary | posterior cingulate gyrus | PCG | Paralimbic |
| inferior frontal gyrus (opercular) | IFGoperc | Association | precentral gyrus | PreCG | Primary |
| inferior frontal gyrus (triangular) | IFGtriang | Association | precuneus | PCUN | Association |
| inferior occipital gyrus | IOG | Association | rolandic operculum | ROL | Association |
| inferior parietal lobule | IPL | Association | superior frontal gyrus (dorsal) | SFGdor | Association |
| inferior temporal gyrus | ITG | Association | superior frontal gyrus (medial) | SFGmed | Association |
| insula | INS | Paralimbic | superior occipital gyrus | SOG | Association |
| lingual gyrus | LING | Association | superior parietal gyrus | SPG | Association |
| middle cingulate gyri | MCG | Paralimbic | superior temporal gyrus | STG | Association |
| middle frontal gyrus | MFG | Association | supplementary motor area | SMA | Association |
| middle occipital gyrus | MOG | Association | supramarginal gyrus | SMG | Association |
| middle temporal gyrus | MTG | Association | temporal pole (middle) | TPOmid | Paralimbic |
| olfactory | OLF | Paralimbic | temporal pole (superior) | TPOsup | Paralimbic |
